# Determinants of COVID-19 Vaccine Hesitancy During the Pandemic: A Cross-Sectional Survey in the Canton of Vaud, Switzerland

**DOI:** 10.3389/ijph.2022.1604987

**Published:** 2022-09-29

**Authors:** Caroline Veys-Takeuchi, Semira Gonseth Nusslé, Sandrine Estoppey, Claire Zuppinger, Julien Dupraz, Jérôme Pasquier, Vincent Faivre, Renzo Scuderi, Sophie Vassaux, Murielle Bochud, Valérie D’Acremont

**Affiliations:** ^1^ Department of Epidemiology and Health Systems, Center for Primary Care and Public Health (Unisanté), Lausanne, Switzerland; ^2^ Swiss Tropical and Public Health Institute (Swiss TPH), Basel, Switzerland

**Keywords:** vaccine hesitancy, COVID-19, pandemic, vaccination, Switzerland

## Abstract

**Objectives:** COVID-19 vaccine hesitancy is a major obstacle in the fight against the pandemic. This study aimed to identify the local determinants of vaccine hesitancy in the context of COVID-19 to better inform future immunization campaigns.

**Methods:** The study, conducted in February 2021, included 1,189 randomly selected inhabitants of the canton of Vaud, Switzerland. Online questionnaires investigated determinants of the intention to vaccinate. Previously validated scores (Cronbach’s alphas >0.70) were applied to our data for inclusion in the ordinal logistic regression model.

**Results:** Individuals were more likely to vaccinate if they were 40 years or older, wealthy, reported a high educational attainment, or reported comorbidities. Doubts regarding vaccine safety and efficacy, mistrust in authorities and a propensity for natural immunity were identified as the main local hindrances to the COVID-19 vaccination.

**Conclusion:** Outreach to people at risk of severe COVID-19 is particularly relevant in the pandemic context to help mitigate vaccine hesitancy in the canton of Vaud, and should take into consideration the level of education. Further investigation is needed to better understand reasons for mistrust in authorities.

## Introduction

In the 2 years since the WHO declared COVID-19 a “Public Health emergency of international concern,” more than 5 million COVID-related deaths have been recorded worldwide [1]. Despite the seriousness of the situation, vaccine hesitancy towards the newly developed vaccines stands in the way of overcoming the pandemic. As illustrated in an Israeli study that presented promising results following rapid vaccine implementation in early 2021, vaccination is currently our strongest tool to achieve this [2–4]. In fact, immunization in general is considered to be one of the most cost-effective interventions to improve health outcomes worldwide [5, 6]. Unfortunately, vaccine hesitancy ranks among the top ten threats to global health in 2019, according to the World Health Organization (WHO) [7]. Identification of the determinants of vaccine hesitancy is therefore imperative to ensure and improve vaccine uptake [6]. A systematic review published in 2018 showed that vaccine acceptance is strongly associated with different levers of trust, depending on the vaccine itself, the health care system and various external factors [8]. Trust in the government, family members and friends’ opinion, as well as historical and socio-cultural factors have also been reported to significantly influence vaccine uptake [8, 9]. Nevertheless, vaccine hesitancy is a complex phenomenon. It is greatly influenced by spatio-temporal factors and by vaccines’ characteristics. There is no universal approach to address it, and so context-specific research must be conducted to inform public health interventions at the local level [10].

In the context of a pandemic, vaccine hesitancy and trust may be further influenced in either directions due to concerns about the safety and efficacy of vaccines that have been rapidly developed, the fear of the new emerging disease and its outcome, and information overload [11]. According to global surveys from early in the pandemic, the overall trust in COVID-19 vaccines increased between November 2020 and January 2021 from 40% to over 50%, with up to 71.5% of the population in high income countries reporting that they would be very, or somewhat, likely to take the vaccine if proven safe and effective [9, 11, 12]. As of January 2022, 78% of the population in high and upper middle income countries had received at least one dose of vaccine, 71% in Switzerland [13].

In Switzerland, vaccination coverage is high for childhood infectious diseases and even showed an upward trend in the last few years [14–17]. Despite these encouraging observations, a large-scale study conducted between 2015 and 2019 listed Switzerland among the five countries in the world with the least confidence in vaccines [18]. Moreover, routine vaccination programs and their implementation vary across cantons, depending on the level of government involvement, among others [14, 19]. Finally, general practitioners play an important role regarding vaccination acceptance as they are considered to be a trust-worthy source of information [14]. Access to high quality scientific information is a critical limit to vaccination in high-income countries and it is also the most frequent reason given by the Swiss population for not getting vaccinated [19]. Surveys from the grey literature on COVID-19 vaccine hesitancy in the Swiss population prior to the vaccine’s availability show diverging results [20, 21]. For example, a survey conducted in September 2020 suggested that more than 50% of the Swiss population planned to be vaccinated once the vaccine was available. Another one conducted in November 2020 that included 40,000 citizens found that 28% of surveyed adults planned to refuse vaccination, while 47% were hesitant. The limited success of the vaccination campaign in Switzerland demonstrates that vaccine skepticism remains a considerable barrier, although there is a lack of reliable Swiss data concerning reasons for vaccine acceptance, hesitancy or refusal during the course of the pandemic.

This study therefore sought to investigate potential sources of vaccine hesitancy in the canton of Vaud, Switzerland in the context of the COVID-19 pandemic, with the aim to develop appropriate and region-specific public health strategies to optimize COVID-19 vaccine uptake. We hypothesized that the level of trust in the authorities, the level of education and a current trend towards natural medicine are amongst the largest barriers to vaccine uptake.

## Methods

### Design and Context

We investigated COVID-19 vaccine hesitancy in the context of a 3rd sero-epidemiological cross-sectional, population-based study in the canton of Vaud, Switzerland (SerocoViD) [22]. The study took place from January 18th to February 6th^,^ 2021. It was part of the national research group Corona Immunitas, launched in March 2020 by the Swiss School of Public Health for the specific purpose of the COVID-19 pandemic and involving scientists from 14 Swiss universities [23]. The primary aim of SerocoViD was to determine the seroprevalence of SARS-CoV-2 in the population.

### Study Population

A random sample of 4,458 residents in the canton of Vaud were identified by the Swiss Federal Statistical Office. Inclusion criteria included: residency in the Canton of Vaud, and aged 15 years and older at study inclusion. Exclusion criteria were: suffering from any cognitive impairment or limitation that would prevent an individual from understanding the aim of the study and answering the questions (e.g., language barrier or being institutionalized). Participants were sampled through a randomly age-stratified method, using five age strata (15-<20; 20-<40; 40-<65; 65-<75; ≥75 years old).

### Procedures

Participants were invited *via* letter including a personal access code; individuals that agreed to take part in the study signed a written informed consent and used their personal code to access online registration for the study visit. In case of study-related questions or technical issues, a hotline service was available. Online questionnaires were available from January 18th to February 6th^,^ 2021. For participants that encountered difficulties in filling out the online questionnaires, study staff were available during the study visit to assist them. Study visits took place in one center located in Lausanne between February 1st and February 6th 2021, during which a locally developed serological test was administered, a Luminex assay detecting anti-SARS-CoV-2 IgG and IgA antibodies [24]. In case participants were unable to attend the study visit on-site, a home visit was possible by means of four mobile study teams. All data were collected and managed using the Research Electronic Data Capture (REDCap) tools hosted at *Unisanté*. REDCap is a secure, web-based software platform designed to support data capture for research studies [25].

### Questionnaires and Variables

Four harmonized questionnaires were elaborated by the national research group Corona Immunitas; the majority of questions can be found in its published protocol [23]. Vaccine-related questions were ultimately designed based on the 5A taxonomy [26], the Global Vaccine Confidence Index [27] and the Vaccine Trust Indicator [28] that were used and validated by previous scientific work [9, 12, 29, 30]. Certain additional items were created by Corona Immunitas members from Ticino. All questionnaires were translated into four languages (French, German, Italian and English) by native speakers. They were reviewed before submission, to avoid errors or technical issues. Questionnaires were auto-administered and covered the following topics: socio-demographic factors; general health; COVID-19-related outcomes; perceptions and behaviors; and vaccination. The vaccination topic included twenty-two close-ended questions, four of which aimed at investigating vaccine hesitancy specifically, using 5-point Likert scales. This has been previously described by Marta Fadda et al. in the Corona Immunitas counterpart results from Canton Ticino [31].

The main outcome of interest of the present study was the intention to vaccinate assessed using a 5-point Likert scale that reflected the participants’ degree of agreement with the statement. Twenty-six potential determinants of vaccine hesitancy were investigated as explanatory variables, similarly assessed using a 5-point Likert scale (Table 1). Previously, Fadda, et al. explored the underlying latent constructs and structure of these same items by means of an Exploratory Factor Analysis that yielded four scores with a Cronbach’s alpha >0.70 [31]. These scores were calculated in our data by summing each item’s score multiplied by its corresponding loading, as reported in Fadda, et al. Two items had a loading factor <0.30 and were thus excluded from the score construct. The items, which investigate the level of trust in authorities and institutions based on the Vaccine Acceptance Index [28], were assessed individually. Age, sex, average monthly household income, level of education and preexisting comorbidities were considered as potential confounders of the main associations. Pre-existing comorbidities included diabetes, immunological disorder (not vaccine-related), hay fever, cardiovascular disease, cancer or past history of cancer, hypertension, respiratory disease and any other chronic disease.

**TABLE 1:** Classification of 26 suggested determinants of vaccine hesitancy into 6 categories, SerocoViD (Vaud, Switzerland, 2021).

Trust in institutions
I generally trust vaccine manufacturers or pharmaceutical companies^a^
I generally trust the Federal Office of Public Health (FOPH)^a^
I understand how vaccination helps my body fight infectious diseases^a^
I feel it is important that I get vaccinated^b^
**Wait and see**
I prefer to wait before being vaccinated until more is known about how effective the vaccine is*^b^ ^,^ ^c^
I prefer to wait before being vaccinated until more is known about the vaccine’s safety*^b^ ^,^ ^c^
I am afraid of possible side effects^d^
**Protect and move on**
I want to protect myself^e^
I want to contribute to the protection of my community/society^e^
I want to contribute to the protection of someone I know who is vulnerable^e^
I want to get back to a normal life as fast as possible^e^
**Preference for alternatives**
I prefer natural immunity against the coronavirus to vaccine-induced immunity^d^
I prefer natural or traditional remedies to the disease rather than being vaccinated^d^
I would rather protect myself by other means (physical distancing, hand hygiene, wearing a mask) than be vaccinated^f^
The coronavirus vaccine has been developed too quickly^c^
**Confidence in protection**
I believe that the vaccination protects me against a severe course of coronavirus infection^e^
I believe that the vaccination protects against transmission of the coronavirus to others^e^
I think that the vaccine will provide long-lasting immunity^f^
**External and medical drivers** ^**^
I am concerned about getting infected if I go to a clinic where vaccinations are administered^f^
Medical reasons (e.g. allergies) prevent me from being vaccinated^e^
I follow what my religious faith prescribes regarding this vaccination^b^ ^,^ ^d^
I base my vaccination decision on the results of my serological test^f^
I am afraid of injections^d^
Non-categorized***
I feel overwhelmed by information on the coronavirus vaccine^c^
I believe that the vaccination protects me against infection with the coronavirus^e^
I prefer to let those who will benefit most have first access to the vaccine^f^

a Ellingson, MK, Sevdalis, N, Omer, SB, and Thomson, A. Validation of the Vaccine Trust Indicator (VTI) in a Multi-Country Survey of Adult Vaccine Attitudes. (unpublished document).

b Larson HJ, et al. Measuring vaccine confidence: introducing a global vaccine confidence index. PLoS Curr. 2015 Feb25;7:ecurrents.outbreaks.ce0f6177bc97332602a8e3fe7d7f7cc4. doi: 10.1371/currents.outbreaks.ce0f6177bc97332602a8e3fe7d7f7cc4. PMID: 25789200; PMCID: PMC4353663.

c Fadda M, Albanese E, Suggs LS. When a COVID-19 vaccine is ready, will we all be ready for it? Int J Public Health. 2020 Jul;65(6):711–712. doi: 10.1007/s00038-020-01404-4. Epub 2020 Jun 11. PMID: 32529534; PMCID: PMC7288619.

d Neumann-Böhme S, et al. Once we have it, will we use it? A European survey on willingness to be vaccinated against COVID-19. Eur J Health Econ. 2020 Sep;21(7):977–982. doi: 10.1007/s10198-020-01208-6. PMID: 32591957; PMCID: PMC7317261.

e Ève Dube, et al. Vaccine Hesitancy, Acceptance, and Anti-Vaccination: Trends and Future Prospects for Public Health. Annual Review of Public Health. Vol. 42:175–191 (Volume publication date April 2021). doi: 10.1146/annurev-publhealth-090419-102240.

f Newly developed item.

*The responses to these items have been reversed for building the scores.

**Cronbach’s alpha <0.70.

***Factor loadings below 0.30.

### Statistical Analyses

Analyses were conducted using R (version 4.0.2) [32]. Results were weighted to account for age stratification in the sampling. As the amount of missing data was less than 10% for each variable, data were not imputated. We generated a radar plot to present the average degree of agreement for each of the 22 explanatory variables, stratified by age, using fmsb and RColorBrewer packages in R. Scores were calculated for each category of those explanatory variables, based on Fadda’s Exploratory Factor Analysis applied to our data. Scores are presented as bar plots and box plots for categories of the vaccine uptake’s determinants. Bivariate relationships between the scores and the intention to vaccinate were assessed using Welch two sample t-tests. Ordinal logistic regression models were used to assess the intention to vaccinate. A first one assessed the effect of age, sex, presence of one or more comorbidities and average monthly income on the intention to vaccinate. The second one investigated the level of education instead of the average monthly income. Both education and income variables were considered in separate models because they were highly correlated. The third one additionally assessed the effect of the constructed categories of vaccine determinants on the intention to vaccinate. Likelihood ratio tests were used to ensure that inclusion of individual covariates improved the model fit (*p*-value < 0.20). The level of trust in authorities and institutions was investigated separately, with the association with the intention to vaccinate presented by means of bar plots. The corresponding bivariate relationships were assessed using chi-squared tests.

### Ethical Considerations

The study protocol was approved by the Cantonal Ethics Committee of Canton de Vaud (CER-VD), Switzerland on April 23, 2020 (ref. 2020 00887). Aside from learning about an individual’s serological status and contributing to scientific knowledge about COVID-19, there was no additional benefit from participating in the study. No financial compensation was provided to study participants with the exception of transport fees to reach the study site, which were reimbursed.

## Results

### Sample Description

Overall, 1,189 participants aged 15–93 years agreed to take part in the study and completed the online questionnaires, corresponding to a participation rate of 26.4%. We collected blood from 1,072 study participants (90.2%), 9.8% (N = 105) *via* a home visit. Participants who had received at least one dose of COVID-vaccine at baseline (N = 59, 4.8%) were excluded from the analyses as their intention to vaccinate was not investigated. Roughly half of participants were female (51.9%), reflecting the original distribution of the invited sample (Supplementary Table S1). In contrast, individuals aged 40 to 64 (26.2%), and 65 to 74 years (26.1%) were overrepresented in comparison to the original invited sample (21.0% and 18.0%, respectively), and individuals aged 20 to 39 (21.2%), and 75 years and older (9.5%) were underrepresented in comparison to the original invited sample (24.1% and 22.2%, respectively). Participants were generally wealthy, with 70% having a net monthly income of at least 6000 CHF. More than half (54.6%) had a high-level of education (i.e., bachelor, advanced or university degree). Finally, the majority of participants were Swiss (81.3%) and in good health.

### Intention to Vaccinate

Overall, 59.4% of unvaccinated participants recorded a score of at least 4/5 with regard to their intention to take the vaccine when made available to them. In multivariable analyses, the intention to vaccinate significantly increased with increasing age category (test for trend = *p* < 0.001) (Table 3). The intention to vaccinate was also significantly associated with a net monthly income of 12,000 CHF and above, educational attainment of university degree, and having at least one comorbidity. Gender was not associated with the intention to vaccinate.

### Determinants of the Intention to Vaccinate

#### Trust in Institutions

**FIGURE 1:**
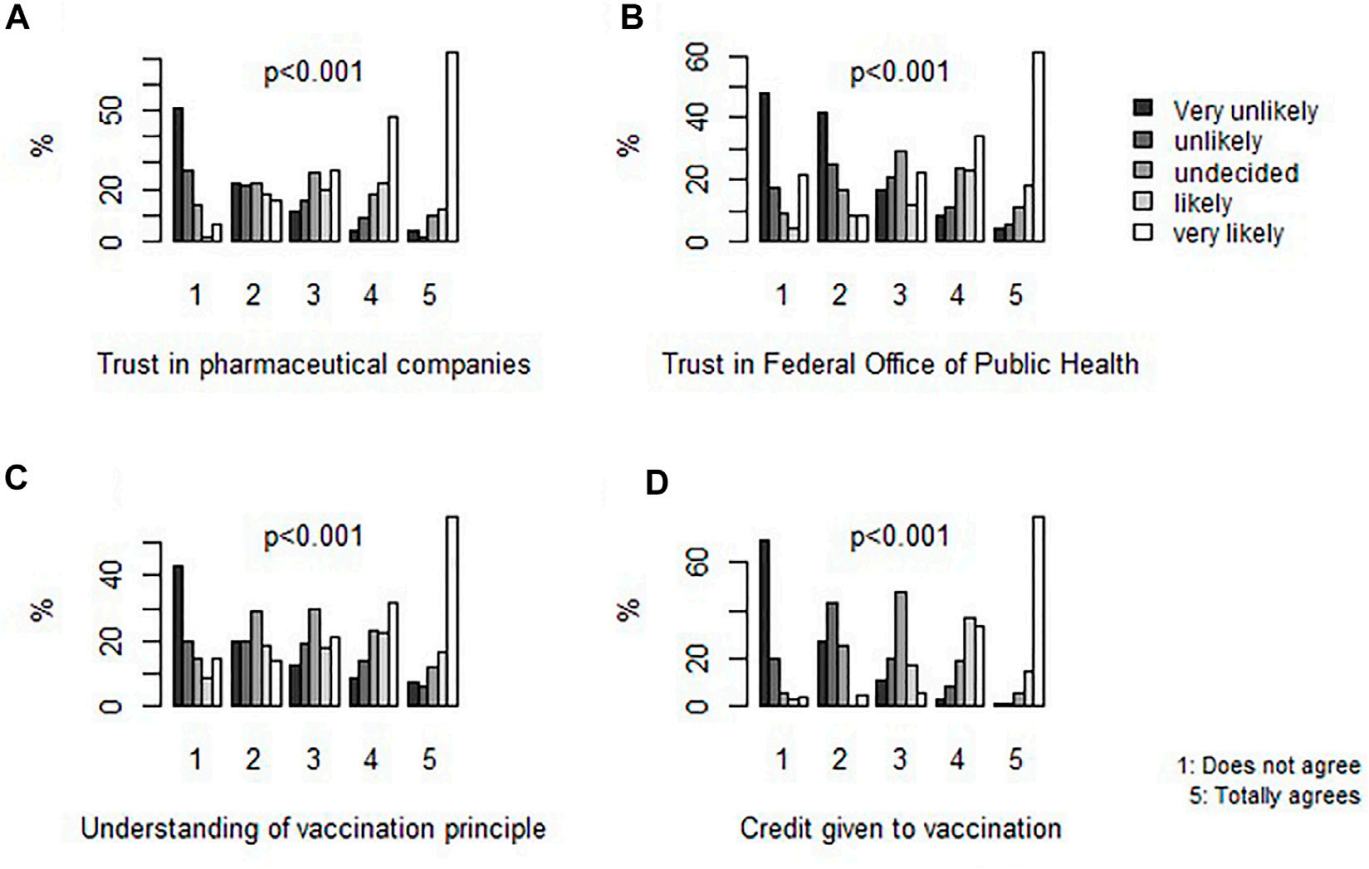
Frequency of intention to vaccinate by level of agreement with each determinant of the Trust-in-institutions category, SerocoViD (Vaud, Switzerland, 2021). Note: *p*-values are derived from chi-squared tests. N = 1,130.

Table 2 shows the distribution of trust in authorities and in institutions according to the intention to vaccinate. Trust in pharmaceutical companies and in the Federal Office of Public Health (FOPH) significantly increased with increasing intention to vaccinate (*p* < 0.001) (Figure 1). Participants that reported understanding vaccination mechanisms and importance were also most likely to vaccinate (*p* < 0.001).

**TABLE 2:** Distribution of the Trust-in-institutions’ items, total and by intention to vaccinate, SerocoViD (Vaud, Switzerland, 2021).

Variable	Number likely to vaccinate (%)					Total
	Very unlikely (N = 116)	Unlikely (N = 129)	Undecided (N = 214)	Likely (N = 206)	Very likely (N = 465)	(N = 1,130)
Trust pharma industry						
Very low	32 (27.6)	17 (13.2)	9 (4.2)	1 (0.5)	4(0.9)	63 (5.6)
Low	24 (20.7)	23 (17.8)	24 (11.2)	19 (9.2)	17 (3.7)	107 (9.5)
Intermediate	34 (29.3)	48 (37.2)	81 (37.9)	59 (28.6)	82 (17.6)	304 (26.9)
High	17 (14.7)	37 (28.7)	76 (35.5)	97 (47.1)	204 (43.9)	431 (38.1)
Very high	9 (7.8)	3 (2.3)	22 (10.3)	27 (13.1)	158 (34.0)	219 (19.4)
NA	0 (0.0)	1 (0.8)	2 (0.9)	3 (1.5)	0 (0.0)	6 (0.5)
Trust in FOPH						
Very low	11 (9.5)	4 (3.1)	2 (0.9)	1 (0.5)	5 (1.1)	23 (2.0)
Low	20 (17.2)	12 (9.3)	8 (3.7)	4 (1.9)	4 (0.9)	48 (4.2)
Intermediate	30 (25.9)	38 (39.5)	54 (25.2)	21 (10.2)	41 (8.8)	184 (16.2)
High	37 (31.9)	49 (38.0)	103 (48.1)	102 (49.5)	152 (32.7)	443 (39.2)
Very high	18 (15.5)	24 (18.6)	46 (21.5)	78 (37.9)	260 (55.9)	426 (37.7)
NA	0 (0.0)	2 (1.6)	1 (0.5)	0 (0.0)	3 (0.6)	6 (0.5)
Understand vaccination						
Not at all	15 (12.9)	7 (5.4)	5 (2.3)	3 (1.5)	5 (1.1)	35 (3.1)
Rather No	13 (11.2)	13 (10.1)	19 (8.9)	12 (5.8)	9 (1.9)	66 (5.8)
More or less	18 (15.5)	28 (21.7)	44 (20.6)	26 (12.6)	31 (6.7)	147 (13.0)
Rather Yes	30 (25.9)	48 (37.2)	80 (37.4)	78 (37.9)	108 (23.2)	344 (30.4)
Yes	40 (34.5)	33 (25.6)	64 (29.9)	87 (42.2)	310 (66.7)	534 (47.3)
NA	0 (0.0)	0 (0.0)	2 (0.9)	0 (0.0)	2 (0.4)	4 (0.4)
Vaccination is important						
Not at all	57 (49.1)	16 (12.4)	4 (1.9)	2 (1.0)	3 (0.6)	82 (7.3)
Rather No	25 (21.6)	40 (31.0)	23 (10.7)	0 (0.0)	4 (0.9)	92 (8.1)
More or less	23 (19.8)	43 (33.3)	105 (49.1)	38 (18.4)	12 (2.6)	221 (19.6)
Rather Yes	7 (6.0)	24 (18.6)	54 (25.2)	104 (50.5)	96 (20.6)	285 (25.2)
Yes	4 (3.4)	5 (3.9)	22 (10.3)	61 (29.6)	347 (74.6)	439 (38.8)
NA	0 (0.0)	1 (0.8)	6 (2.8)	1 (0.5)	3 (0.6)	11 (1.0)

NA, no answer.

#### Wait and See

**FIGURE 2:**
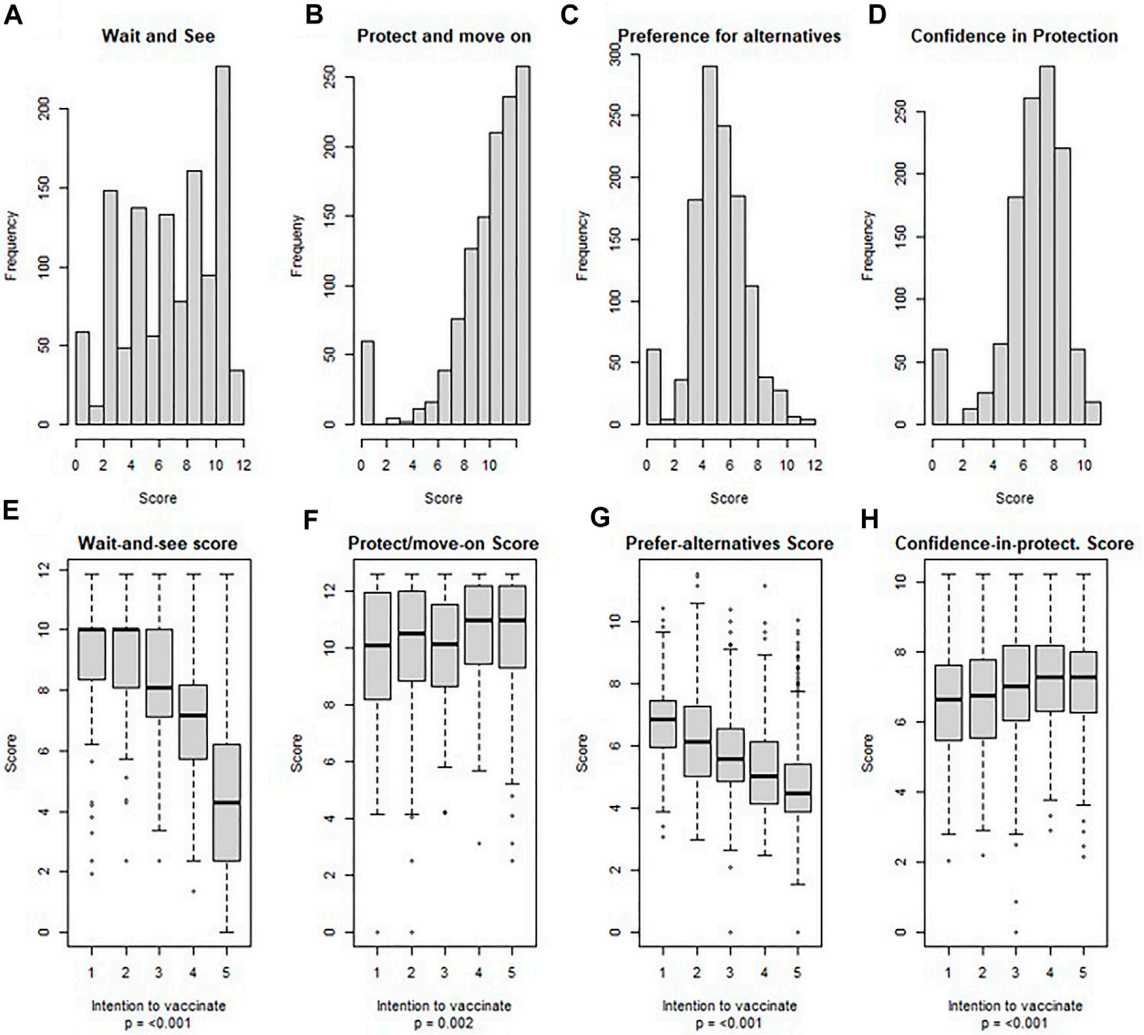
Scores for categories of vaccine uptake’s determinants, crude and by intention to vaccinate, SerocoViD (Vaud, Switzerland, 2021). Note: Scores were calculated based on previous Exploratory Factor Analysis by Fadda, et al. applied to our data; N = 1,130.

Figure 2 depicts the scores for categories of vaccine uptake, crude and by intention to vaccinate (on a 5-Likert scale). A higher desire to wait-and-see before getting vaccinated was significantly associated with a lower intention to vaccinate (*p* < 0.001). In multivariable regression analysis, those with more doubts about the security and efficacy of the vaccines were 42% less likely to consider vaccination (*p* < 0.001) (Table 3).

**TABLE 3:** Ordinal logistic regression analysis for the intention to vaccinate, SerocoViD (Vaud, Switzerland, 2021).

	Model 1				Model 2				Model 3			
	OR	2.5% CI	97.5% CI	*p*-value	OR	2.5% CI	97.5% CI	*p*-value	OR	2.5% CI	97.5% CI	*p*-value
Gender (female)	0.84	0.66	1.07	0.15	0.80	0.64	0.99	**0.05**	1.08	0.83	1.40	0.58
Age 20-39^a^	1.42	0.97	2.09	0.07	1.02	0.66	1.58	0.93	1.48	0.98	2.23	0.06
Age 40-64^a^	1.59	1.09	2.30	**0.02**	1.32	0.87	2.02	0.19	1.76	1.17	2.65	**0.007**
Age 65-74^a^	3.80	2.54	5.71	**<0.001**	3.02	1.95	4.70	**<0.001**	3.76	2.39	5.95	**<0.001**
Age ≥75^a^	6.65	3.87	11.67	**<0.001**	4.33	2.51	7.56	**<0.001**	4.49	2.42	8.46	**<0.001**
Comorbidity (yes)^b^	1.34	1.06	1.71	**0.02**	1.22	0.97	1.54	0.08	1.33	1.02	1.73	**0.04**
Income 3000-5999 CHF/month	0.95	0.62	1.45	0.82					0.77	0.49	1.22	0.27
Income 6000-8999 CHF/month	1.22	0.81	1.85	0.34					0.87	0.55	1.36	0.53
Income 9000-11999 CHF/month	1.64	1.05	2.56	**0.03**					1.11	0.69	1.80	0.67
Income ≥12000 CHF/month	3.06	1.99	4.71	**<0.001**					1.75	1.10	2.81	**0.02**
Professional training^c^					0.87	0.58	1.32	0.51				
Matura or vocational baccalaureate^c^					1.06	0.70	1.59	0.79				
Higher technical college^c^					0.96	0.62	1.48	0.84				
University studies^c^					2.82	1.82	4.39	**<0.001**				
Wait and see^d^									0.58	0.55	0.62	**<0.001**
Protect and move on^d^									1.27	1.18	1.36	**<0.001**
Preference for alternatives^d^									0.77	0.70	0.83	**<0.001**
Confidence in Protection^d^									1.36	1.24	1.49	**<0.001**
AIC^e^	2807.52	3164.30	2257.94									

a Ref.: age 15–19.

b ≥1 chronic conditions: Immunological, cardio-vascular, respiratory, hypertension, diabetes, non-vaccine related allergy, cancer, other chronic condition.

c Ref.: no school certificate.

d Factorial analysis scores based on Fadda, et al.

e AIC , akaike information criterion.

#### Protect and Move on

**FIGURE 3:**
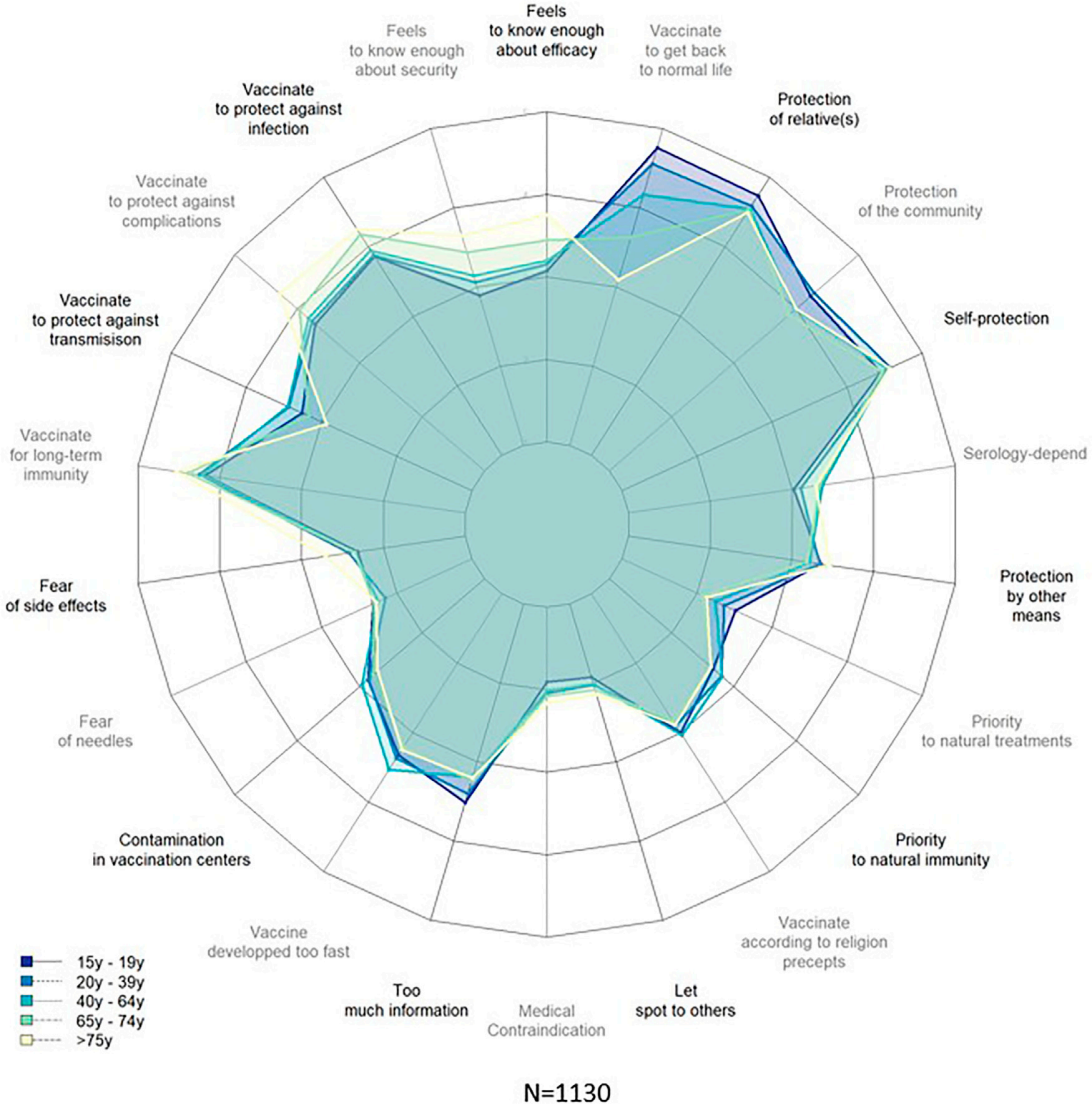
Degree of agreement with potential determinants of vaccine uptake, stratified by age category, SerocoViD (Vaud, Switzerland, 2021).

A high score was reached regarding participants’ wishes to protect themselves, their community and vulnerable relatives (Figure 2). These results were similar for those who do not or rather not intend to vaccinate. The unadjusted effect size of that category on the intention to vaccinate was small, yet significant (*p* = 0.002). Figure 3 shows a radar plot for the degree of agreement with 22 determinants of vaccine uptake, stratified by age category. Younger individuals were particularly keen to get back to a normal life. Participants aged 15 to 19 and 20 to 39 years old were also particularly willing to protect their community (84.3% and 89.2% respectively). In the multivariable regression analysis, the odds of intention to vaccinate was 27% higher among those who were most willing to protect themselves and the community (*p* < 0.001) (Table 3).

#### Preference for Alternatives

The score relative to participants’ preference for alternatives was normally distributed, and negatively associated with the intention to vaccinate (*p* < 0.001) (Figure 2). In the multivariable regression, individuals who reported being most in favor of alternatives to vaccination, such as traditional remedies, natural immunity or physical distancing, and those who thought that the vaccine had been developed too quickly, had a 23% lower odds of intention to vaccinate (*p* < 0.001) (Table 3).

#### Confidence in Protection

The score relative to the confidence that the vaccine protects against infection and a severe course of disease, and that it provides a long lasting immunity was generally high (Figure 2). The unadjusted association between confidence-in-protection and intention to vaccinate was small, yet significant (*p* < 0.001). After adjustment for potential confounders, the odds of intention to vaccinate was 36% higher (*p* < 0.001) among those who reported being most confident in vaccine protection (Table 3).

## Discussion

This study investigated potential sources of COVID-19 vaccine hesitancy at an early stage of the Swiss vaccination campaign during the pandemic. Although the intention to vaccinate was high, it was not at the level now known to be required to avoid hospital overloads. Willingness to vaccinate was higher among wealthy and highly educated people, among those aged 40 years and older and those with one or more comorbidities. Despite the willingness of young people to protect their community, their limited risk to develop severe complications and die from COVID-19, together with their doubts about the security and efficacy of the COVID-19 vaccines, potentially contributed to young, healthy participants being less willing to vaccinate. This is consistent with prior literature [9, 12, 33]. It is also known that people who have never experienced infectious diseases that are now managed and under control by virtue of vaccination (e.g., polio or tetanus), are less willing to get vaccinated [34–36]. Although the intention to vaccinate was higher among older age groups, this study found that 22.4% of people aged 40–64 years, and 13.4% of those aged 65 years and older were unwilling to vaccinate at the start of the vaccination campaign. This demographic constituted a reservoir of potentially severely ill patients, sufficiently large to reach the critical point of hospital capacity.

This study also found that participants did not believe in the vaccines’ ability to reduce viral transmission. This could be due to the health authorities and media coverage of COVID-19 vaccine breakthrough cases, which did not always highlight the generally good level of protection by vaccines against severe disease or death [37]. However, at the time the survey was conducted, little was known regarding the effect of the COVID-19 vaccines on viral transmission. It is now believed that they reduce onward transmission *via* their efficiency at preventing infection. Importantly, although vaccine effectiveness against transmission of the Delta variant and Omicron variant is reduced in comparison to the Alpha variant, vaccines still efficiently prevent hospitalizations and death [38–40].

Individuals that trust health authorities and pharmaceutical institutions are more likely to get vaccinated, as corroborated by Lazarus et al. in their “Global survey of potential acceptance of COVID-19 vaccine” [9]. On one hand, trust in the vaccines’ ability to prevent infection and a feeling of responsibility for one another’s safety were also significant drivers of the intention to vaccinate in the canton of Vaud. On the other hand, this study found that the lack of hindsight on the vaccines’ safety and efficacy, and a preference for natural methods were the main barriers towards vaccination. The preference for natural immunity is related to a lack of trust in the vaccines and the fear of what is going to be injected. It might be reinforced by a general propensity of the Swiss population for alternative medicines, and their more general naturalistic vision of the world and the body (Freikörperkultur). These more general opinions and views of health have been previously shown to be associated with a reluctance to vaccinate [14, 41, 42]*.*

By the time the COVID-19 certificate was established in Switzerland on September 13th, 2021, 80.4% of individuals aged 15 years and older were vaccinated with at least one dose [43], which is more than projected by the results of the present study as of February 6th, 2021 (4.8% vaccinated; 59.4% with intention to vaccinate). The percentage of vaccinated individuals was higher than anticipated among individuals aged 40 to 64 (82.4% compared to the estimated 56.4%), 65 to 74 (89.4% compared to the estimated 69.8%), and 75 years and older (91.5% compared to the estimated 81.3%). This suggests that the vaccination campaigns conducted between February and September 2021 may have reached people aged 40 and older that were initially undecided or unlikely to vaccinate once the vaccine was available.

### Strengths and Limitations of the Study

An important strength of the SerocoViD survey is the random selection of participants from the general population. Moreover, there was limited missing data, permitting all analyses to be conducted without requiring imputation. In addition, the determinants of vaccine hesitancy included in the survey were varied and investigated through validated methods and scales. However, a potential limitation of the current results and their generalizability is the low participation rate, which resulted in the age distribution being non-representative of the original sample population, with a particular overrepresentation of middle aged people. Similarly, low participation rate have been reflected in other Swiss-based population surveys that included blood sampling [44]. Study participation rates may have been influenced by the fact that the study visit was only available at a single location for the entire canton of Vaud, therefore the travel distance may have selectively discouraged those living in remote places as well as middle-aged active people to participate. In an attempt to improve participation rates, on-site assistance with filling-in the online questionnaires and home visits were provided as an alternative to on-site visits. Moreover, the promise of getting to know their serological status possibly encouraged participants to take part in the survey out of curiosity. Another potential limitation of the present study is the potential bias of results due to the overall high educational attainment of the study sample. Lastly, given the cross-sectional nature of the present study, causal inference with regards to the observed associations was not possible.

### Conclusion

The present study identified various determinants of vaccine hesitancy at an early stage in the course of the pandemic. Results from this study also support the need to apply public health interventions that take into account individuals with a lower educational attainment and for which various tools have previously been suggested [9, 45–47]. Moreover, in order to address vaccine hesitancy, it is critical to investigate the root causes of mistrust among Swiss people with regards to authority as it is a well-known key of success of vaccination campaigns [9, 45]. A considerable proportion of people at risk of severe COVID-19 were reluctant to vaccinate at the time the survey was conducted. In the context of the pandemic, Swiss public health measures should be particularly focused on that, albeit minority, part of vaccine hesitant people in order to reduce the burden on the Swiss health care system.

Overall, the findings of the present study are in line with prior international literature. It allowed to refine the whys and wherefores of vaccine hesitancy in the canton of Vaud in the pandemic context. It may give a valuable insight to our local public health authorities to optimize their approach in dealing with future pandemic situations. Further in-depth, possibly qualitative, research is necessary to better apprehend the causes of the above-identified determinants of vaccine hesitancy and give additional context-specific tools to deal with them.
