# Supplementary material for: Determinants of COVID-19 Vaccine Hesitancy During the Pandemic: A Cross-Sectional Survey in the Canton of Vaud, Switzerland
Source: Int J Public Health. 2022 Sep 29;67:1604987. doi: 10.3389/ijph.2022.1604987 (PMC9556695; doi:10.3389/ijph.2022.1604987)
Supplement: Supplementary file 1 [file Table1.docx]

**Table S1: Descriptive characteristics of the data set, SerocoViD (Vaud, Switzerland, 2021):**

| **Variable** | **Number likely to vaccinate (%)** | | | | | **Total** |
| --- | --- | --- | --- | --- | --- | --- |
|  | **Very unlikely**  **(N=116)** | **Unlikely**  **(N=129)** | **Undecided**  **(N=214)** | **Likely**  **(N=206)** | **Very likely**  **(N=465)** | **(N=1130)** |
| Sex |  |  |  |  |  |  |
| Men | 52 (44.8) | 54 (41.9) | 89 (41.6) | 101 (49.0) | 248 (53.3) | 544 (48.1) |
| Women | 64 (55.2) | 75 (58.1) | 125 (58.4) | 105 (51.0) | 217 (46.7) | 586 (51.9) |
|  |  |  |  |  |  |  |
| Age category (years) |  |  |  |  |  |  |
| 15-19 | 31 (26.7) | 32 (24.8) | 45 (21.0) | 40 (19.4) | 44 (9.5) | 192 (17.0) |
| 20-39 | 24 (20.7) | 38 (29.5) | 51 (23.8) | 53 (25.7) | 74 (15.9) | 240 (21.2) |
| 40-64 | 35 (30.2) | 31 (24.0) | 63 (29.4) | 54 (26.2) | 113 (24.3) | 296 (26.2) |
| 65-74 | 20 (17.2) | 24 (18.6) | 45 (21.0) | 41 (19.9) | 165 (35.5) | 295 (26.1) |
| ˃74 | 6 (5.2) | 4 (3.1) | 10 (4.7) | 18 (8.7) | 69 (14.8) | 107 (9.5) |
|  |  |  |  |  |  |  |
| Net monthly income (CHF) (CHF(((CHF)(CHF/month) |  |  |  |  |  |  |
| < 3000 | 20 (17.2) | 12 (9.3) | 33 (15.4) | 19 (9.2) | 37 (8.0) | 121 (10.7) |
| 3000-5999 | 30 (25.9) | 26 (20.2) | 45 (21.0) | 42 (20.4) | 84 (18.1) | 227 (20.1) |
| 6000-8999 | 31 (26.7) | 28 (21.7) | 52 (24.3) | 50 (24.3) | 111 (23.9) | 272 (24.1) |
| 9000-11999 | 13 (11.2) | 21 (16.3) | 31 (14.5) | 31 (15.0) | 72 (15.5) | 168 (14.9) |
| ≥ 12000 | 6 (5.2) | 22 (17.1) | 28 (13.1) | 40 (19.4) | 115 (24.7) | 211 (18.7) |
| NA*^1^* | 16 (13.8) | 20 (15.5) | 25 (11.7) | 24 (11.7) | 46 (9.9) | 131 (11.5) |
|  |  |  |  |  |  |  |
| Highest level of education |  |  |  |  |  |  |
| Compulsory education | 28 (24.1) | 31 (24.0) | 49 (22.9) | 41 (19.9) | 60 (12.9) | 209 (18.5) |
| Diploma | 34 (29.3) | 38 (29.5) | 59 (27.6) | 47 (22.8) | 119 (25.6) | 297 (26.3) |
| Bachelor’s degree | 17 (14.7) | 15 (11.6) | 36 (16.8) | 30 (14.6) | 49 (10.5) | 147 (13.0) |
| Advanced vocational school | 26 (22.4) | 26 (20.2) | 31 (14.5) | 45 (21.8) | 79 (17.0) | 207 (18.3) |
| University degree | 10 (8.6) | 16 (12.4) | 38 (17.8) | 41 (19.9) | 154 (33.1) | 259 (22.9) |
| NA | 1 (0.9) | 3 (2.3) | 1 (0.5) | 2 (1.0) | 4 (0.9) | 11 (1.0) |
| Nationality |  |  |  |  |  |  |
| Swiss | 94 (81.0) | 100 (77.5) | 179 (83.6) | 172 (83.5) | 370 (79.6)  6 | 915 (81.0) |
| Non-Swiss | 22 (19.0) | 29 (22.5) | 35 (16.4) | 34 (16.5) | 95 (20.4) | 215 (19.0) |
|  |  |  |  |  |  |  |
| Smoking habits |  |  |  |  |  |  |
| Current smoker | 20 (17.2) | 26 (20.2) | 34 (15.9) | 35 (17.0) | 70 (15.1) | 185 (16.4) |
| Ex-smoker | 15 (12.9) | 19 (14.7) | 45 (21.0) | 49 (23.8) | 132 (28.4) | 260 (23.0) |
| Never-smoker | 80 (69.0) | 82 (63.6) | 134 (62.6) | 121 (58.7) | 259 (55.7) | 676 (59.8) |
| NA | 1 (0.9) | 2 (1.6) | 1 (0.5) | 1 (0.5) | 4 (0.9) | 9 (0.8) |
|  |  |  |  |  |  |  |
| Body Mass Index (Kg/m2) |  |  |  |  |  |  |
| ˂18.5 | 12 (10.3) | 5 (3.9) | 11 (5.1) | 8 (3.9) | 11 (2.4) | 47 (4.2) |
| 18.5-24.9 | 66 (56.9) | 77 (59.7) | 128 (59.8) | 114 (55.3) | 233 (50.1) | 618 (54.7) |
| 25-29.9 | 23 (19.8) | 31 (24.0) | 50 (23.4) | 64 (31.1) | 150 (32.3) | 318 (28.2) |
| 30-34.9 | 10 (8.6) | 9 (7.0) | 17 (7.9) | 13 (6.3) | 55 (11.8) | 104 (9.2) |
| 35 | 3 (2.6) | 2 (1.6) | 4 (1.9) | 6 (2.9) | 9 (1.9) | 24 (2.1) |
| NA | 2 (1.7) | 5 (3.9) | 4 (1.9) | 1 (0.5) | 7 (1.5) | 19 (1.6) |
|  |  |  |  |  |  |  |
| Hypertension |  |  |  |  |  |  |
| Yes | 5 (4.3) | 11 (8.5) | 29 (13.6) | 27 (13.1) | 99 (21.3) | 171 (15.1) |
| No | 110 (94.8) | 116 (89.9) | 185 (86.4) | 179 (86.9) | 365 (78.5) | 955 (84.5) |
| NA | 1 (0.9) | 2 (1.6) | 0 (0.0) | 0 (0.0) | 1 (0.2) | 4 (0.4) |
|  |  |  |  |  |  |  |
| Diabetes |  |  |  |  |  |  |
| Yes | 2 (1.7) | 3 (2.3) | 5 (2.3) | 9 (4.4) | 27 (5.8) | 46 (4.0) |
| No | 113 (97.4) | 124 (96.1) | 209 (97.7) | 197 (95.6) | 437 (94.0) | 1080 (95.6) |
| NA | 1 (0.9) | 2 (1.6) | 0 (0.0) | 0 (0.0) | 1 (0.2) | 4 (0.4) |
|  |  |  |  |  |  |  |
| Cardiovascular disease |  |  |  |  |  |  |
| Yes | 1 (0.9) | 4 (3.1) | 5 (2.3) | 14 (6.8) | 47 (10.1) | 71 (6.2) |
| No | 114 (98.3) | 123 (95.3) | 209 (97.7) | 192 (93.2) | 417 (89.7) | 1055 (93.4) |
| NA | 1 (0.9) | 2 (1.6) | 0 (0.0) | 0 (0.0) | 1 (0.2) | 4 (0.4) |
|  |  |  |  |  |  |  |
| Respiratory disease respiratoryredisedisease |  |  |  |  |  |  |
| Yes | 10 (8.6) | 4 (3.1) | 16 (7.5) | 12 (5.8) | 43 (9.2) | 85 (7.5) |
| No | 105 (90.5) | 123 (95.3) | 198 (92.5) | 194 (94.2) | 421 (90.5) | 1041 (92.1) |
| NA | 1 (0.9) | 2 (1.6) | 0 (0.0) | 0 (0.0) | 1 (0.2) | 4 (0.4) |
|  |  |  |  |  |  |  |
| History of cancer |  |  |  |  |  |  |
| Yes | 1 (0.9) | 1 (0.8) | 7 (3.3) | 2 (1.0) | 23 (4.9) | 34 (3.0) |
| No | 114 (98.3 ) | 126 (97.7) | 207 (96.7) | 204 (99.0) | 441 (94.8) | 1092 (96.6) |
| NA | 1 (0.9) | 2 (1.6) | 0 (0.0) | 0 (0.0) | 1 (0.2) | 4 (0.4) |
|  |  |  |  |  |  |  |
| Immunological disorder |  |  |  |  |  |  |
| Yes | 4 (3.4) | 3 (2.3) | 6 (2.8) | 11 (5.3) | 23 (4.9) | 47 (4.2) |
| No | 111 (95.7) | 124 (96.1) | 208 (97.2) | 195 (94.7) | 440 (94.6) | 1078 (95.4) |
| NA | 1 (0.9) | 2 (1.6) | 0 (0.0) | 0 (0.0) | 2 (0.4) | 5 (0.4) |
|  |  |  |  |  |  |  |
| Hay fever |  |  |  |  |  |  |
| Yes | 32 (27.6) | 25 (19.4) | 52 (24.3) | 56 (27.2) | 109 (23.4) | 274 (24.2) |
| No | 83 (71.6) | 102 (979.1) | 162 (75.7) | 150 (72.8) | 355 (76.3) | 852 (75.4) |
| NA | 1 (0.9) | 2 (1.6) | 0 (0.0) | 0 (0.0) | 1 (0.2) | 4 (0.4) |
|  |  |  |  |  |  |  |
| Comorbidities^2^ |  |  |  |  |  |  |
| ≥ 1 | 41 (35.3) | 42 (32.6) | 86 (40.2) | 94 (45.6) | 237 (51.0) | 500 (44.2) |
| 0 | 75 (64.7) | 87 (67.4) | 128 (59.8) | 112 (54.4) | 228 (49.0) | 630 (55.8) |

*^1^NA = No answer*

*^2^≥1 chronic conditions: Immunological, cardio-vascular, respiratory, hypertension, diabetes, non-vaccine related allergy, cancer, other chronic condition.*
